# Research hotspots and trends in unexplained recurrent spontaneous abortion (URSA) from 2014 to 2024: a bibliometric analysis

**DOI:** 10.3389/fmed.2025.1554875

**Published:** 2025-05-14

**Authors:** Yishi Jiang, Yan Zhang, Yuyan Li, Yan Che

**Affiliations:** NHC Key Lab of Reproduction Regulation, Shanghai Engineering Research Center of Reproductive Health Drug and Devices, Shanghai Institute for Biomedical and Pharmaceutical Technologies, Shanghai, China

**Keywords:** unexplained recurrent spontaneous abortion (URSA), spontaneous abortion, habitual abortion, women health, bibliometric analysis, CiteSpace, VOSviewer, visual analysis

## Abstract

**Introduction:**

Recurrent spontaneous abortion (RSA) represents a significant challenge in obstetrics and reproductive medicine. Causative factors in 40%–50% of RSA couples remain unknown, a condition termed unexplained recurrent spontaneous abortion (URSA). This study employed bibliometric analysis to elucidate global research trends and identify key areas of interest in URSA.

**Methods:**

We utilized the Web of Science Core Collection (WoSCC), including Science Citation Index Expanded (SCI-EXPANDED) and Social Sciences Citation Index (SSCI), as our data source. Our search encompassed all publications on URSA published between 1 January 2014 and 30 October 2024. Following rigorous removal of duplicates, we retained 586 relevant publications, including 532 original articles and 54 reviews. We conducted bibliometric analysis using CiteSpace, VOSviewer and Microsoft Excel.

**Results:**

Analysis of annual publications and their citations demonstrated significant growth over the last 10 years. China, the United States and Iran emerged as the most productive countries in the field. Author distribution indicated the absence of a cohesive core author group, suggesting a dispersed research community. The top five cited publications included one prospective observational study, one randomized controlled trial, two reviews, and one immunohistochemistry study, focusing on the etiology, interventions, and therapies of URSA. Keyword cluster analysis identified six categories, with the top three keywords being “expression,” “polymorphisms,” and “regulatory T-cells.”

**Conclusion:**

This bibliometric analysis reveals three key research domains over the last decade in URSA: immunological mechanism and therapies, genetic mechanism, and anticoagulation therapies. While these areas have advanced our understanding, limitations persist in etiological heterogeneity and therapeutic inconsistencies. Future studies should prioritize rigorous multicenter trials with phenotypic stratification, and multi-omics approaches for mechanistic insights. Enhanced global collaboration and interdisciplinary integration are essential to transition from empirical management to evidence-based precision medicine in URSA.

## 1 Introduction

Recurrent spontaneous abortion (RSA) is associated with increased risks of adverse pregnancy outcomes including preterm labor, low birth weight, and other obstetric complications. The condition also significantly impacts patients’ psychological wellbeing, making it a major challenge in reproductive medicine. The World Health Organization (WHO) defines RSA as the occurrence of three or more consecutive miscarriages before the 20th week of gestation. In China, RSA is defined by expert consensus (1) as two or more consecutive miscarriages with the same partner before the 28th week of gestation.

The estimated incidence of RSA ranges from 1% to 5% (2–4), with an increased risk of recurrence associated with the number of previous miscarriages. The etiology of RSA is complex and highly heterogeneous, involving chromosomal abnormalities, immune dysfunction, pre-thrombotic states and uterine anatomical factors. Nevertheless, causative factors in 40%–50% of RSA couples remain unknown. It is referred as unexplained recurrent spontaneous abortion (URSA) (5) or idiopathic recurrent spontaneous abortion (6). Most studies have indicated that URSA may be associated with genetic and immunological factors (7, 8). However, there is considerable variation in specific genetic or immunological pathways that researchers have identified, with different conclusions in the same topic. Additionally, some researchers have explored the potential mechanisms of URSA through empirical treatments.

Given these knowledge gaps, it is crucial to understand current global research trends and key areas of interest in URSA. Bibliometric analysis provides a valuable approach to evaluate research performance, analyze knowledge maps, and identify emerging trends in this field. Our study employs this methodology to systematically assess the current research landscape and predict future directions.

## 2 Materials and methods

### 2.1 Data source and search strategy

We conducted our analysis using the Web of Science Core Collection (WoSCC), a high-quality literature database (9, 10) and continuous updates to provide reliable information. To ensure methodological rigor, we specifically utilized two of its major indices Science Citation Index Expanded (SCI-EXPANDED) and Social Sciences Citation Index (SSCI).

We performed a comprehensive search using the following search query: TS = “unexplained recurrent spontaneous abortion” or “unexplained recurrent pregnancy loss” or “unexplained recurrent miscarriage” or “unexplained habitual abortion” or “idiopathic recurrent spontaneous abortion” or “idiopathic recurrent pregnancy loss” or “idiopathic recurrent miscarriage” or “idiopathic habitual abortion” (6). We limited our search to Article or Review Article, published in English between 1 January 2014 and 30 October 2024. After removing duplicate documents, we obtained 586 publications (532 articles and 54 reviews).

### 2.2 Data statistics and indicators

We performed bibliometric visualization and analysis using CiteSpace software (version 6.3.R1; Drexel University, Philadelphia, Pennsylvania, United States) (11) and VOSviewer software (version 1.6.20; Centre for Scientific and Technological Research, Leiden University, Leiden, Netherlands) (12). We also used Microsoft Excel for quantitative analysis. The analyzed indicators included the number of publications, average citations per publication, and the distribution of countries, institutions and authors of the papers, as well as keyword analysis. In the network diagram, different nodes represent different institutions, countries/regions, or authors. The size of the circles corresponds to the number of publications, with larger circles indicating a higher number of publications.

## 3 Results

### 3.1 The number of publications and citation frequency

Our analysis included 586 publications, consisting of 532 articles and 54 reviews. A total of 3,136 authors from 911 institutions across 56 countries contributed to the publications in 209 journals, with 16,568 references sourced from 2,894 journals.

**FIGURE 1:**
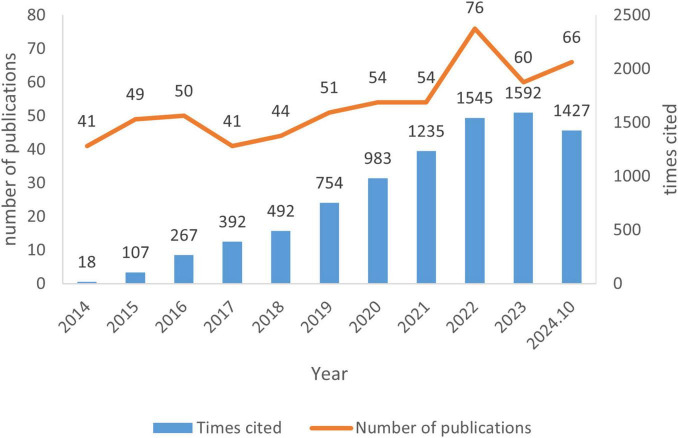
The number of publications and citation frequency in the field of unexplained recurrent spontaneous abortion (URSA) between 1 January 2014 and 30 October 2024.

The annual publication count increased from 41 in 2014 to a peak of 76 in 2022, followed by a decline to 60 in 2023 (Figure 1). These publications received 8,814 total citations (average 15.04 citations/publication), with peak annual citations reaching 1,592 in 2023. Citation frequency demonstrated significant growth over the past decade, as detailed in Figure 1.

### 3.2 Distribution of countries/regions

**FIGURE 2:**
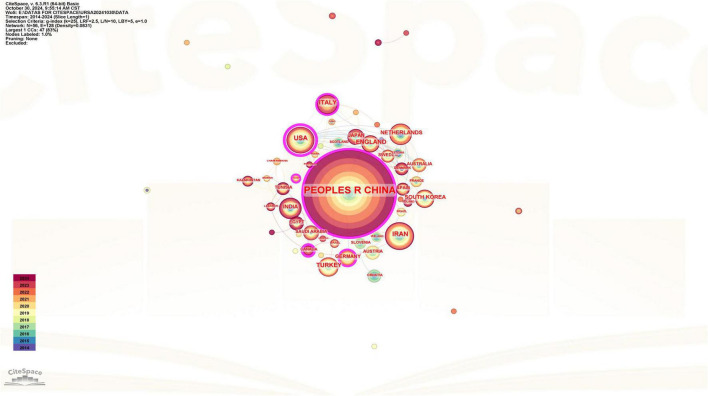
Network map of unexplained recurrent spontaneous abortion (URSA) research co-countries/regions in the Web of Science Core Collection (WoSCC) database. There are 56 nodes, which means 56 countries/regions included, 128 nodes connections, and the network density is 0.0831.

China emerged as the most productive country with 307 publications, averaging 12.24 citations per article. The United States followed with 41 publications, averaging 31.80 citations per article, and Iran came third with 38 publications, averaging 15.66 citations per article. One of the principal metrics employed by CiteSpace is betweenness centrality, which indicates the proximity of research collaborations between countries or regions. Generally, a value of 0.1 or higher is considered a satisfactory cut-off value for this centrality. The United States topped the list with a betweenness centrality of 0.64, followed by Canada at 0.31, China at 0.26, Italy and Germany, both at 0.16. The thickness of the purple outer circle as visualized in Figure 2 indicated the strength of research collaboration between countries.

There was a discrepancy between publication volume and betweenness centrality. While China dominated productivity, it didn’t have the highest betweenness centrality. The United States, with less than a seventh of China’s publications, had the highest betweenness centrality. Iran, with a publication count close to the United States, had its centrality subthreshold (< 0.1). This indicated that China and Iran should strengthen their international research networks.

Among the institutions, Shanghai Jiao Tong University, Sun Yat-sen University, Shandong University, Fudan University, and Sichuan University published the most publications (Table 1). It is worth noting that none of these institutions had a betweenness centrality exceeding 0.1.

**TABLE 1:** The top five number of publications in institutions in the field of unexplained recurrent spontaneous abortion (URSA).

Rank	Institution	Number of publications	Times cited	Average times cited
1	Shanghai Jiao Tong University	27	386	14.30
1	Sun Yat-sen University	27	364	13.48
3	Shandong University	21	200	9.52
4	Fudan University	20	357	17.85
5	Sichuan University	15	180	12.00

### 3.3 High-influence authors and author collaborations

The analysis encompassed 3,136 authors, averaging 5.35 authors per document. Nam Keun Kim led with the highest number of publications, publishing 13 publications with an average of 13.46 citations per article, primarily focusing on genetic polymorphisms and URSA. His highly cited work discussed miR-27a and miR-449b polymorphisms (13). Aimin Zhao ranked second with 11 publications and an average of 14.27 citations per article. His most cited publication was a meta-analysis of lymphocyte immunotherapy for URSA (14). Junhao Yan contributed 10 publications, averaging 8.40 citations per article. His most cited work analyzed the loss pattern in couples with chromosomal abnormalities compared to those with unexplained repeated miscarriages (15).

In terms of citation impact, Jian Li, Bo Xu, Camille Sylvestre achieved the highest total citations with 258, 190, and 177 citations, respectively. A group including Bouet et al. (17) stood out with the highest average citation count, receiving 173 citations. Nam Keun Kim had the highest H-index of eight among all authors. Notably, all authors exhibited low betweenness centrality values (< 0.01), reflecting limited collaborative connections.

According to Price’s law (16), approximately half of the publications in the same field are written by a group of highly productive authors, whose number is approximately equivalent to the square root of the total number of authors. The number of publications published by core authors was 0.749*n_*max*_^0^.^5^ = 2.70. Based on this number, we identified three or more authors as core authors. The 174 core authors published 288 publications, accounting for 49.15% of the total. This figure was close to the predicted half of the total publications. However, the absolute number of core authors exceeded the square root of the total number of authors, indicating that authors in this field were dispersed without formation of a stable core research group.

### 3.4 Highly cited literature

Highly cited publications, which are pivotal in the field, are recognized for their important research results. Table 2 summarized the essence of the top-cited works by detailing the titles, methodologies, first author, publication years, and the associated journals’ impact factor over the last 5 years, conclusions, and the frequency with which these works have been cited. The table illustrated the diverse methodologies and topics of the top five highly cited studies. Two of these publications primarily delved into the etiology of URSA. The top cited article observed chronic endometritis in women with URSA (17), while the other characterized uterine Natural killer (NK) cells using immunohistochemistry techniques (18). The remaining studies explored interventions or therapies for URSA. Among these, one RCT examined the role of progesterone (19), a review assessed the clinical use of corticosteroids (20), and a Cochrane review focused on Aspirin and/or heparin (21). Two of these publications appeared in highly regarded journals with an IF = 10 (NEW ENGL J MED, Hum Reprod Update).

**TABLE 2:** The top five highly cited documents on unexplained recurrent spontaneous abortion (URSA) in the Web of Science Core Collection (WoSCC) database.

Publication title	Research method*	First author	Year	Journal	Conclusion	Citation
Chronic endometritis in women with recurrent pregnancy loss and recurrent implantation failure: prevalence and role of office hysteroscopy and immunohistochemistry in diagnosis (17)	A prospective observational study	Pierre-Emmanuel Bouet	2016	Fertil Steril (7.5)	A high prevalence of immunohistochemically confirmed chronic endometritis (CE) was found in women with recurrent implantation failure and unexplained recurrent pregnancy loss.	173
A randomized trial of progesterone in women with recurrent miscarriages (19)	A multicenter, double-blind, placebo-controlled, randomized trial	Arri Coomarasamy	2015	New Engl J Med (94.3)	Progesterone therapy in the first trimester of pregnancy did not result in a significantly higher rate of live births among women with a history of unexplained recurrent miscarriages.	171
The clinical use of corticosteroids in pregnancy (20)	A narrative review of the scientific literature	M W Kemp	2016	Hum Reprod Update (17.2)	There is both significant scope and an urgent need for further research-informed refinement to the use of antenatal corticosteroids in pregnancy.	151
Aspirin and/or heparin for women with unexplained recurrent miscarriage with or without inherited thrombophilia (21)	A systematic Review	Paulien G de Jong	2014	Cochrane Database Syst Rev (9.9)	The effect of anticoagulants in women with URSA and inherited thrombophilia needs to be assessed in further randomized controlled trials; at present there is no evidence of a beneficial effect.	133
Characterization of uterine NK cells in women with infertility or recurrent pregnancy loss and associated endometriosis (18)	Immunohisto-chemistry	Emma Giuliani	2014	Am J Reprod Immunol (3.2)	Women, with or without endometriosis, who have larger populations of cytotoxic CD16(+) uNK cells and/or higher populations of NKp46(+) CD56(+) cells may be at greater risk of infertility disorders resulting from an inflammatory environment occurring during implantation or later during decidualization.	108

*A prospective observational study: a non-interventional cohort study design that follows participants over time to analyze relationships between exposures and outcomes without manipulating variables. A multicenter, double-blind, placebo-controlled, randomized trial: A gold-standard clinical trial conducted across multiple sites, using random allocation, blinding of participants/investigators, and placebo comparison to rigorously evaluate therapeutic efficacy. A narrative review of the scientific literature: a critical qualitative synthesis of existing evidence on a topic, offering interpretative insights without structured methodology or statistical pooling. A systematic review: a reproducible, PRISMA-guided evidence synthesis that systematically identifies, appraises, and synthesizes all eligible studies on a focused research question, often incorporating meta-analytic techniques. Immunohisto-chemistry: a histopathological technique employing antigen-antibody interactions to detect and localize specific proteins in tissue sections, visualized via enzymatic or fluorescent labeling.

### 3.5 Research hotspots and frontiers

#### 3.5.1 Keyword visualization

After exclusion of direct search terms (e.g., “miscarriage,” “unexplained recurrent spontaneous abortion”) and application of a minimum occurrence threshold of five, we identified 185 keywords. The most frequently keywords were “expression” (121 occurrences), “polymorphisms” (67 occurrences), “regulatory T-cells” (63 occurrences), “natural killer cells” (60 occurrences), “risk” (47 occurrences), “association” (45 occurrences), “cells” (43 occurrences), “peripheral blood” (41 occurrences), “cytokines” (40 occurrences), and “meta-analysis” (38 occurrences).

**FIGURE 3:**
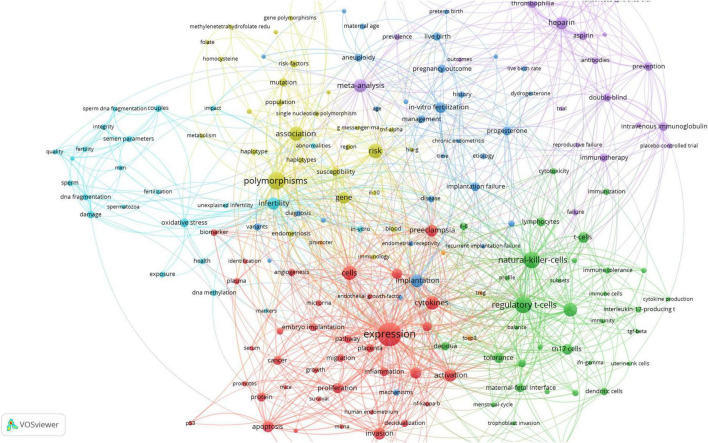
Keywords visualization in unexplained recurrent spontaneous abortion (URSA) research field. Colors mean clusters; circles mean frequencies; lines mean associations.

Visual clustering revealed six dominant research themes (Figure 3): (1) Expression, activation, cells, cytokines (cluster 40 keywords); (2) Risk, polymorphism, association, gene (cluster 28 keywords); (3) Regulatory T-cells, NK cells, IL, HLA (cluster 34 keywords); (4) Heparin, aspirin, thrombosis prevention, experimental studies, meta-analysis (cluster 25 keywords); (5) Implantation, implantation failure, *in vitro* fertilization, live births, progesterone (cluster 31 keywords); (6) Infertility, sperm, DNA fragmentation, oxidative stress (cluster 23 keywords).

#### 3.5.2 Burst keywords

The dynamic nature of keywords is reflected in their frequency fluctuations over periods ranging from one to several years. This phenomenon offers insights into areas of significant interest, such as emerging trends and frontiers in knowledge. By analyzing the top 14 burst keywords and their duration period (Table 3), polymorphism and anticoagulation therapy were the main topics in the early period of 2014–2015. An increase in studies focusing on immune mechanisms, particularly T cell related studies, was observed after 2018. In recent years, starting from 2021 to 2022, studies targeting the mechanisms of URSA, including cell migration and gene expression, emerged with progress in gene science. Currently, ongoing research focused on keywords such as maternal-fetal interface, migration, invasion, promotes, and protein.

**TABLE 3:** Top 14 keywords with the strongest citation bursts.

Keywords	Strength	Begin	End
Polymorphisms	2.91	2014	2019
Disease	2.79	2014	2018
Variants	2.67	2014	2015
Aspirin	2.65	2015	2017
Early pregnancy	2.68	2016	2018
Differentiation	3.56	2017	2021
Receptors	2.90	2017	2018
Th17 cells	3.11	2018	2021
Treg cells	2.62	2018	2020
Maternal-fetal interface	2.56	2020	2024
Promotes	2.63	2021	2024
Invasion	2.57	2021	2024
Migration	3.72	2022	2024
Protein	2.60	2022	2024

## 4 Discussion

We conducted a bibliometric analysis of URSA research using data from the Web of Science Core Collection (SCI-EXPANDED and SSCI). The results showed a growing interest in URSA, with research concentrating on etiological mechanisms and potential therapies.

### 4.1 Global achievements

China, the United States, and Iran lead in terms of publication outputs. However, when considering centrality and the citation frequency, the United States stood out for its strong international cooperation, exerting significant influence. This advantage likely stems from well-established research infrastructure and substantial funding support, particularly for genetic studies of URSA. Despite high productivity, researchers from China and Iran showed relatively lower academic impact, suggesting the need for enhanced investments and international collaborations.

Our author analysis indicated that core authors significantly contributed to URSA research field, but a stable core group was not formed. Researchers from South Korea and China were among the most productive, yet not the most frequently cited. The absence of betweenness centrality values exceeding 0.01 indicated weak collaborative networks among researchers. This likely reflected the complex nature of URSA, involving multiple factors and stages, substantial etiological heterogeneity, and diverse therapies.

### 4.2 Research hotspots and trends

Bibliometric analysis of keyword clustering and burst word temporal trends revealed that URSA research over the past decade predominantly investigated three key areas: immunological mechanism and treatment, genetic mechanism, and anticoagulation therapy. The precise etiology of URSA remains unclear due to its diagnosis by exclusion and the absence of standardized reference values. URSA involves a multistage, complex process, potentially involving alterations at the genetic, protein, and metabolic levels, exhibiting considerable etiological heterogeneity (22). Consequently, precision treatments are developed toward diverse avenues.

#### 4.2.1 Immunological mechanisms and treatment

From an immunological perspective, pregnancy is recognized as a semi allograft that escapes rejection, and normal maternal-fetal immune tolerance is a prerequisite for a successful pregnancy. A disruption to this immune tolerance may result in adverse effects on embryo implantation and development, potentially leading to miscarriage (23). Current evidence implies that immune factors are responsible for 60% of URSA cases (24). Immunological etiologies include autoimmune and alloimmune conditions (25), where alloimmune RSA, representing two-thirds of unexplained cases, remains primarily a diagnosis of exclusion.

The bibliometric analysis highlighted three predominant immunological research foci: T cells in adaptive immunity, NK cells in innate immunity, and immunomodulatory therapy. The most focused topics were the imbalance of ratios between Th17 and Treg cells and the ensuing changes in cytokine expression (26–30). Studies have shown elevated Th17 and Th17-related cytokines, and diminished regulatory T cells (Tregs) numbers in URSA patients (26–29). This Th17/Treg cell model provides a framework for understanding URSA pathogenesis that could not be explained by the traditional Th1/Th2 model (30). A great number of studies have been conducted on the genetic factors of T cells. For example, forkhead box protein 3 (Foxp3) is a key transcription factor of Treg, and Foxp3 polymorphisms rs2232365 and rs3761548 may be associated with URSA (31, 32).

Natural killer cell abnormalities and/or dysfunction have been reported to be associated with reproductive disorders. Notably, NK cells exhibit distinct phenotypes and function in peripheral blood (pbNK), endometrium (uNK) and decidua (dNK). It is generally accepted that an increase in the number and activity of pbNK cells correlates with URSA (33). However, some studies have demonstrated no significant difference in pbNK cells between URSA patients and healthy controls (34). While some studies have indicated uNK cells’ involvement in infertility, others have yielded conflicting results (35). In URSA patients, increased cytotoxic CD16(+) uNK cells and/or higher numbers of NKp46(+) CD56(+) cells have been observed, which may foster an inflammatory environment during implantation or decidualization (18). dNK cells in URSA patients displayed aberrant polarization process and persistently elevated cytotoxic reactivity, accompanied by markedly reduced extracellular matrix (ECM) transcription - factors potentially contributing to failed fetal tolerance (36).

Immunomodulatory therapy has also garnered significant interest in recent years. Different approaches have been reported to increase the pregnancy rate and live birth rates in URSA patients, including lymphocyte immunization therapy (LIT) (14, 37, 38), intravenous infusion of immunoglobulin (IVIG) (39, 40), fat emulsion therapy (41), and human amniotic epithelial cells (42). For instance, allogeneic LIT administered before and during pregnancy has shown greater effectiveness compared to administration solely before pregnancy (14). Low-dose LIT has shown the ability to rebalance the peripheral blood Th1/Th2/Treg paradigm (38). IVIG has demonstrated the ability to reduce cytotoxic T cells and NK cells, while increasing immunosuppressive T cells, regulating cytokines, and controlling the immune-inflammatory response (40). However, their efficacy remains controversial (24, 43), necessitating further high-quality studies for validation.

#### 4.2.2 Genetic factors

Genetic factors, including chromosomal polymorphisms, chromosomal abnormalities and genetic abnormalities, play a crucial role in RSA pathogenesis (44). A systematic review identified 13 genes and 21 variants engaged in immune responses (IFNG, IL10, KIR2DS2, KIR2DS3, KIR2DS4, MBL, TNF), coagulation (F2, F5, PAI-1, PROZ), metabolism (GSTT1, MTHFR), and angiogenesis (NOS3, VEGFA) associated with URSA (6). However, study heterogeneity, resulting from varying populations and diagnostic definitions, calls for further research through genome-wide association studies or large-scale studies with identified associations. Whole genome sequencing (WGS) and whole exome sequencing (WES) hold promise in identifying novel causes of pregnancy loss, given the current scarcity of population-based studies (45, 46) and animal studies (47).

MicroRNAs (miRNAs) are small non-coding RNAs that regulate gene expression post-transcriptionally, by hybridizing to target mRNAs. Specific mutations in miRNAs, such as single-nucleotide polymorphisms (SNPs), may disrupt miRNA-mediated regulation. The disruption may result in the development of certain female reproductive dysfunctions (48). miRNAs have shown potential as diagnostic tools for various reproductive disorders (49). However, the association between miRNA polymorphisms and URSA remains underexplored, with inconclusive study results (50–54).

Male factors may contribute to URSA, but the exact association remains largely unknown. Sperm DNA fragmentation (sDF) has emerged as a valuable marker for male fertility, despite some controversies. Higher levels of sDF have been observed in URSA couples compared to fertile controls (55–60). Furthermore, a significant association was reported between increased sDF rate and aberrant methylation of H19/IGF2 and KCNQ1 genes (61). However, a study reported no difference between cases and controls in sDF (62). Reductions in sperm count, quality, and motility may also elevate the risk of URSA (55, 62), although findings were not entirely consistent. Oxidative stress in spermatozoa and seminal plasma represented another potential mechanism (63).

#### 4.2.3 Anticoagulant therapy

Currently, patients with URSA are typically evaluated for thrombophilia susceptibility and treated with low-dose aspirin (LDA), low molecular weight heparin (LMWH), or a combination of the two (44, 64). LDA has been shown to improve endometrial tolerance during the mid-luteal phase in patients with URSA (65). However, a Cochrane review including nine RCTs found insufficient evidence to support anticoagulant use in URSA patients (21). A recent multicenter study has demonstrated that the administration of LMWH and/or LDA during pregnancy serves as an effective intervention in URSA patients, notably improving live birth rates (64). Several studies indicated that the effectiveness of patient subgroups may vary. For instance, benefits have been observed in patients with three or more RSA (66, 67), as well as in getting through the early stage of pregnancy (68, 69). Conversely, no significant benefit has been noted in patients negative for antiphospholipid antibodies (70).

### 4.3 Limitations

Several limitations should be considered when interpreting the findings of this bibliometric analysis. The incomplete annual data for 2024 may influence the accuracy of temporal trend analyses. The restriction of our search to the Web of Science Core Collection (SCI-E and SSCI) databases may have excluded potentially relevant studies not indexed in these resources. Furthermore, English-language inclusion criterion could have resulted in the omission of significant research published in other languages. Finally, the inherent etiological and therapeutic heterogeneity of URSA may introduce potential biases in our interpretation of research trends and patterns.

## 5 Conclusion

This bibliometric analysis reveals three key research domains over the last decade in URSA: immunological mechanism and therapies, genetic mechanism, and anticoagulation therapies. While these areas have advanced our understanding, limitations persist in etiological heterogeneity and therapeutic inconsistencies. Future studies should prioritize rigorous multicenter trials with phenotypic stratification, and multi-omics approaches for mechanistic insights. Enhanced global collaboration and interdisciplinary integration are essential to transition from empirical management to evidence-based precision medicine in URSA.

## Data Availability

The original contributions presented in the study are included in the article/supplementary material, further inquiries can be directed to the corresponding author/s.
